# Combination of Machine Learning Techniques to Predict Overweight/Obesity in Adults

**DOI:** 10.3390/jpm14080816

**Published:** 2024-07-31

**Authors:** Alberto Gutiérrez-Gallego, José Javier Zamorano-León, Daniel Parra-Rodríguez, Khaoula Zekri-Nechar, José Manuel Velasco, Óscar Garnica, Rodrigo Jiménez-García, Ana López-de-Andrés, Natividad Cuadrado-Corrales, David Carabantes-Alarcón, Vicente Lahera, Carlos Hugo Martínez-Martínez, J. Ignacio Hidalgo

**Affiliations:** 1 Department of Computer Architecture, School of Informatic, Universidad Complutense de Madrid, 28040 Madrid, Spain; 2 Public Health and Maternal-Child Health Department, School of Medicine, Universidad Complutense de Madrid, 28040 Madrid, Spain; 3 Health Research Institute of the Hospital Clínico San Carlos (IdISSC), 28040 Madrid, Spain; 4 Physiology Department, School of Medicine, Universidad Complutense de Madrid, 28040 Madrid, Spain; 5 Medicine Department, School of Medicine, Universidad Complutense de Madrid, 28040 Madrid, Spain

**Keywords:** overweight/obesity, machine learning, artificial intelligence, predictive model

## Abstract

(1) Background: Artificial intelligence using machine learning techniques may help us to predict and prevent obesity. The aim was to design an interpretable prediction algorithm for overweight/obesity risk based on a combination of different machine learning techniques. (2) Methods: 38 variables related to sociodemographic, lifestyle, and health aspects from 1179 residents in Madrid were collected and used to train predictive models. Accuracy, precision, and recall metrics were tested and compared between nine classical machine learning techniques and the predictive model based on a combination of those classical machine learning techniques. Statistical validation was performed. The shapely additive explanation technique was used to identify the variables with the greatest impact on weight gain. (3) Results: Cascade classifier model combining gradient boosting, random forest, and logistic regression models showed the best predictive results for overweight/obesity compared to all machine learning techniques tested, reaching an accuracy of 79%, precision of 84%, and recall of 89% for predictions for weight gain. Age, sex, academic level, profession, smoking habits, wine consumption, and Mediterranean diet adherence had the highest impact on predicting obesity. (4) Conclusions: A combination of machine learning techniques showed a significant improvement in accuracy to predict risk of overweight/obesity than machine learning techniques separately.

## 1. Introduction

According to the data reported by the Spanish National Institute of Statistics, in the last 30 years, the prevalence of obesity in Spain has been multiplied by 2.4, changing from 7.4% in 1987 to 17.4% in 2017 [1]. This increased overweight/obesity prevalence is currently considered as global epidemic, which constitutes an important public health problem [2]. According to the World Health Organization, overweight is defined as a condition of excessive fat deposits, while obesity is a chronic complex disease defined by excessive fat deposits that can impair health. The diagnosis of overweight and obesity is performed by measuring people’s weight and height and by calculating their body mass index (BMI): weight (kg)/height^2^ (m^2^). For adults, overweight is a BMI greater than or equal to 25 kg/m^2^ and obesity is a BMI greater than or equal to 30 kg/m^2^ [3].

A large number of risk factors for overweight/obesity has been widely studied [4]. It is widely known that population obesity prevention strategies based on non-specific recommendations for physical activity, healthy diet, and models of healthy social rules have the potential to decrease overweight/obesity levels [5]. However, these approaches have not achieved desirable results, suggesting the existence of biological factors closely associated with the risk of overweight/obesity [6]. Indeed, the classical risk factors for obesity may be classified into modifiable (lifestyle factors and models of healthy social rules) and non-modifiable (sex and age) categories [7,8]. Modifiable and non-modifiable factors should be analyzed together to estimate the risk of developing overweight/obesity. However, it is a significant challenge for researchers and epidemiologists since it is required to use large-scale datasets in which traditional modeling assumptions, such as linearity, lack of multicollinearity, and proportional risk/odds/hazards over time, should not be considered [9]. Interestingly, systems based on artificial intelligence and machine learning have been proposed as an appealing alternative approach for building predictive models [10]. Artificial intelligence can be defined as the development of systems endowed with intellectual processes typical of human beings, such as reasoning, generalization, improvement through past experiences, and the discovery of meanings [11]. Based on different machine learning techniques, several approaches have tried to identify modifiable and non-modifiable risk factors responsible for obesity prevalence variation at the population level [12]. In this regard, inactivity, an improper and unhealthy diet, age, sex, hypertension, diabetes, and sociodemographic aspects have been related to overweight/obesity by different studies using artificial intelligence models [13,14,15,16,17]. However, available studies have evaluated heterogeneous and a limited number of factors, and there is a lack of studies that attempt to jointly analyze non-modifiable biological aspects and modifiable aspects related to lifestyle and health status closely related to obesity using artificial intelligence models. In addition, recent studies have proposed different machine learning methods to predict obesity, in which variables, such as height, weight, and even body mass index, were included as inputs in the datasets of predictive models, thus limiting the predictive power of the proposed models [18,19].

Taking all of these factors together, it is crucial to design optimal predictive models for overweight/obesity without anthropometric measurements that may condition the obvious classification of overweight/obesity with high accuracy. These models would allow us to identify and classify subjects at high risk of developing overweight/obesity in the future, thus enabling us to implement effective preventive risk reduction strategies and, therefore, decreasing the incidence levels of overweight/obesity at the population level. Accordingly, the aim of the present work was to design an algorithm for predicting the risk of overweight/obesity based on different modifiable lifestyle factors and health state as well as non-modifiable biological factors using a cascade classifier flow based on an innovative combination of various classical machine learning algorithms.

## 2. Materials and Methods

### 2.1. Data Source

The present study employed an observational design that collected data from 1179 participants older than 18 years old, who were recruited by 14 recruitment centers, including hospitals and universities in Madrid. Biological and sociodemographic factors as well as aspects related to lifestyle and health state in residents in Madrid (Spain) were anonymously recruited. Different validated questionnaires were administered through a web-based platform. The Mediterranean diet adherence survey (MEDAS test) was used to analyze nutrition habits, while physical activity intensity was analyzed using the International Physical Activity Questionnaire (IPAQ). A total of 38 variables with different categories for each one of them was recorded. Detailed descriptions and categories for these variables are shown in the Supplementary Table S1.

In the inclusion study, all participants accepted and signed an informed consent form. The study was approved by the Regional Clinical Drugs Research Ethics Committee of the Community of Madrid (Comité Ético de la Investigación con Medicamentos Regional de la Comunidad de Madrid CEIm-R, Approval Code: 06/2018. Approval date: 28 June 2018) and conducted in accordance with the Declaration of Helsinki. This project is part of the GenObIA consortium of the Madrid Community (GenObIA-CM.B2017/BMD-3773).

### 2.2. Classical Machine Learning Algorithms and Predictive Model Based on Cascade Classifier Flow

The widely used nine classical machine learning algorithms AdaBoost (ADB), bagging classifier (BC), Bernoulli Naïve Bayes (BNB), decision tree and extra trees (DT and ETs), gradient boosting (GB), Gaussian Naïve Bayes (GNB), logistic regression (LR), and random forest regressor (RFR) [20,21,22] were independently tested using cross-validation. To determine the predictive accuracy of developing overweight/obesity, 75% of the dataset was destined for training the model (training dataset). Once the classifiers were trained, their learning was tested with the remaining 25% of data (test dataset). The output obtained from prediction was compared to actual values of the dataset, obtaining successful percentage levels for each predictive model.

**Figure 1:**
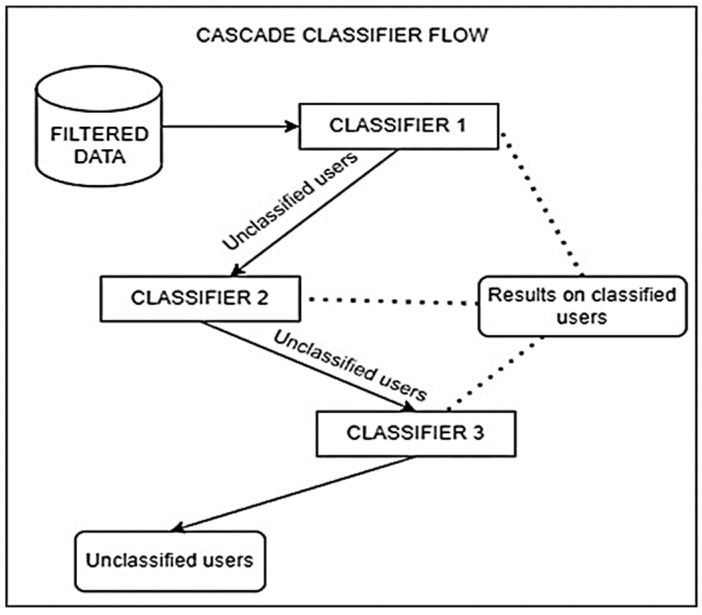
Cascade classifier operation flow. Basis structure of proposed cascade classifier, combining gradient boosting (classifier 1), random forest (classifier 2), and logistic regression (classifier 3) machine learning techniques.

A cascade classifier flow was proposed. It was constituted by the three classifiers with the best results to predict overweight/obesity in the training phase. Overweight/obesity and healthy classification limit thresholds of 70 and 80%, respectively, were considered with the aim of obtaining the highest possible number of successfully classified samples, increasing precision (percentage of correct predictions) and decreasing the misclassification rate. As shown in Figure 1, the best classifier in training proceeds to evaluate the test dataset. The results are classified into two groups: First, the cases that are positively classified according to the established threshold; second, the cases in which the probability of classification does not reach this limit threshold are not classified by the first classifier. The second group was passed to the second classifier and the same process was repeated. The evaluation process was repeated until all classifiers were used or there were no more individuals to be classified.

### 2.3. Performance Assessing Metrics of the Algorithms

Machine learning algorithms for classification are typically evaluated using simple methodologies that will be familiar to many medical researchers and clinicians. In the current study, accuracy, precision, recall, and F1-score evaluation metrics were used to test the performance of predictive algorithms.

Accuracy: measures the proportion of correct predictions from the total number of predictions. It is obtained as the number of correct predictions divided by the total number of predictions [23].

Accuracy = (true positives + true negatives)/total predictions

Precision: a metric that quantifies the accuracy of positive (overweight/obesity) and negative (normal weight) predictions [23].

Precision overweight/obesity = true positives/true positives + false positives (positive prediction value)

Precision normal weight = true negatives/true negatives + false negatives (negative prediction value)

Recall: an important metric used in classifications to evaluate the performance of a model. It measures the model’s ability to correctly identify cases (overweight/obesity) and healthy (normal weight) subjects [23].

Recall overweight/obesity (sensitivity) = true positives/actual positives (true positives + false negatives)

Recall normal weight (specificity) = true negatives/actual negatives (true negatives + false positives)

F1-score: the harmonic mean of precision and recall. It provides a single metric that combines both precision and recall. It is calculated as 2 × (precision × recall)/(precision + recall).

### 2.4. Ranking of the Predictive Algorithm: Statistical Validation

One-hundred runs were launched to compare the predictive capacity of each classical machine learning algorithm and our proposal of the cascade classifier flow. A set of metrics variables, including accuracy (percentage of data correctly classified), misclassification rate (percentage of misclassified data), precision for each class (percentage of correct prediction), sensitivity or positive recall, and specificity or negative recall, was obtained. 

In addition, a non-parametric statistical study based on the Friedman test was performed to detect significant differences between the behavior of two or more algorithms. The Friedman test was used as an analog of the two-way analysis of variance [24,25], but in this case, by range variance. The first step was to convert original predictive results into ranks for each algorithm/instance. Once the information was gathered, ranks were generated as follows: for each instance (i), the values were classified from 1 (best result) to k (worst result, this being the maximum number of algorithms), with each rank defined as:

$$R_{j}=(1\leq j\leq k)$$

Therefore, the rank of the algorithm must be calculated based on the ranks obtained in each instance:

$$R_{j}=\frac{1}{n}\overset{n}{\underset{i=1}{\sum }}r_{i}^{j}$$

The Friedman statistic *F_f_* [26] was computed as

$$F_{f}=\frac{12n}{k\left(k+1\right)}\left[\underset{j}{\sum }R_{J}^{2}-\frac{k{\left(k+1\right)}^{2}}{4}\right]$$

which was distributed according to an X^2^ distribution with k − 1 degrees of freedom. The null hypothesis (H0) considered that all predictive models were equal, being plausible to look at the critical value according to the distribution of X^2^ with 9 degrees of freedom and an α value of 0.05.

### 2.5. Predictive Variables with a Greater Impact on Overweight/Obesity Risk: SHAP Tool

The shapely additive explanation (SHAP) technique was used to determine the impact of the variables on weight gain. SHAP is a theoretic approach for explaining the output of any machine learning model, providing a unified framework that supports various interpretations based on the contribution of each input variable in the model system. SHAP is widely used in the quest for the interpretability and explainability of predictive machine learning models [27,28,29]. This tool has the ability to identify the priority of the contributions of all features from a global perspective, providing a visual and comprehensive approach to increase the transparency of ensemble models, which helps with interpreting and understanding the entire model and with visualizing feature attributions at the observation level for any machine learning model [30].

## 3. Results

### 3.1. Description of Recruited Population

Data from 1179 subjects were recorded to develop predictive models using classical machine learning techniques separately and the cascade classifier flow. The study population showed a balanced distribution between men (48.3%) and women (51.7%), with an average age of 41.21 ± 0.58 years, in the range of 18–69 years. It was observed that 41.8% (567 subjects) was overweight/obese, which supported a sample of a large enough size for the learning of the predictive algorithms used. Detailed distributions of additional variables and categories used as inputs are shown in Supplementary Table S1.

### 3.2. Cross-Validation of Classical Machine Learning Algorithms

A 10 k-fold cross-validation was performed on the nine classical machine learning models. Table 1 shows the precision and deviation obtained after the cross-validation test for each predictive model. The results reveal that the five predictive models, bagging, logistic regression, gradient boosting, extra trees, and random forest, show the highest accuracy values, ranging from 0.69 to 0.72 (Table 1).

### 3.3. Results of the Cascade Classifier Model

Different combinations among classical predictive algorithms were carried out with the aim of creating the best cascade classifier model. It is important to note that weight, height, and body mass index were not included as variables when developing a predictive model. Finally, the three classical models, gradient boosting, random forest, and logistic regression, constituted the cascade classifier. The combination of the above-described classical machine learning algorithms reported the highest accuracy in the test phase compared to the rest of the combinations (80%).

- Gradient boosting was used as the first-level classifier. This model focused on performing the largest individual classification between the high risk of overweight/obesity or normal weight groups. In this case, an accuracy of 80% was obtained out of a total of 135/295 classified individuals. The false-negative value was low (18), indicating that the recall of the overweight/obese class was 89% since there were hardly any cases of individuals suffering from overweight/obesity. Unclassified subjects were passed to the classification model of the following level.
- Random forest was used as the second-level classifier. The number of classified individuals was smaller than gradient boosting since the input data of this model were unclassified individuals by gradient boosting. The level of difficulty of classification increases as the cascade classifier progresses. However, 34 individuals out of the 160 received were successfully classified. The results of the confusion matrix are quite good since the false-negative value was still low (3), with a recall of almost 70%.
- Logistic regression was used as the third-level classifier. This classifier classified the fewest individuals, since their factors did not clearly express to the model any type of classification within the established levels. The results reveal that the other 24 individuals were successfully classified with an accuracy of 83% and an excellent positive recall of 93%, since only one false negative was obtained. This means that, of the 15 overweight cases that had entered this model, only one of them was wrongly classified as non-overweight.

Table 2 shows the precision level, numbers of classified and unclassified individuals, and the confusion matrix for predicting both classes (normal weight and overweight/obesity) of the proposed cascade classifier model. Initially, all recruited variables (38) were considered in the cascade flow model, obtaining an accuracy of 80% and successfully classifying 65.5% of the total number of tested individuals (Table 2). The precision and recall variables for both classes are around 80%, suggesting that the numbers of false negatives and positives are practically null. In addition, we decided to perform additional tests by eliminating several variables to determine how they might affect the predictive ability of the cascade model. In this regard, age and recruitment center variables were removed from the predictive model, generating two alternative instances with 37 variables. The age variable was removed since it is a continuous non-modifiable variable that is closely related to overweight/obesity. In this line of reasoning, it would be plausible to think that the university recruitment center provided the majority of data from young people, which could be considered to introduce a certain level of bias. For this reason, we also decided to eliminate this variable, using a model with 37 variables without a recruitment center (37c). The results obtained with 37 variables without age (37a) or without a recruitment center (37c) achieved a classification rate close to the value obtained with 38 variables (instance 38) (Table 2). However, the predictive model based on 38 variables not only achieved the highest value of a correct classification rate and total number of classified subjects, but also the lowest number of unclassified numbers (Table 2).

### 3.4. Comparison of Effectiveness between Classical Machine Learning Algorithms and Cascade Classifier for Predicting Overweight/Obesity

Different metrics variables were measured to compare the predictive capacity of the cascade classifier with respect to the rest of the classical machine learning algorithms. Table 3 shows the variables related to the accuracy, precision, and recall of predictions. The results reveal that the cascade classifier obtained the best results for all variables compared to the classical predictive models, with almost 80% of the data being correctly classified and the highest values of precision and recalls with a very low false-positive rate. These results show that the cascade flow model is the most effective model for predicting overweight/obesity, showing the highest values of accuracy as well as precision and recall for both normal-weight and overweight/obesity groups (Table 3). In order to eliminate bias due to age or recruitment center, we tested the ability to predict the overweight/obesity of the cascade classifier using a dataset with only 36 variables (without age or a recruitment center), revealing the cascade flow model was also the most effective model for predicting overweight/obesity (Supplementary Table S2). This suggests the robustness of the proposed model.

**Figure 2:**
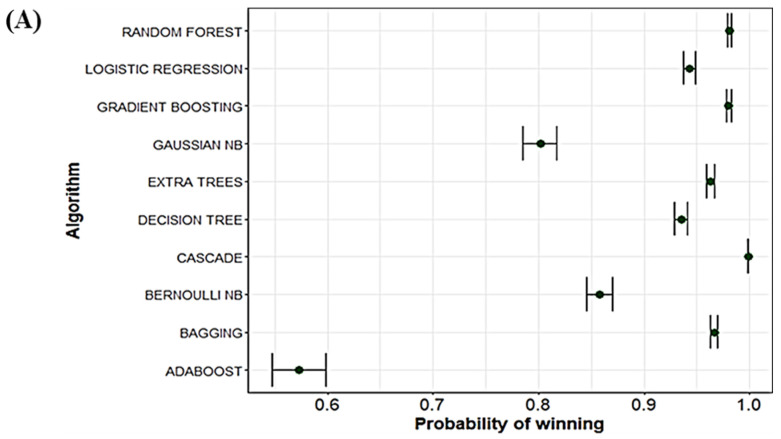

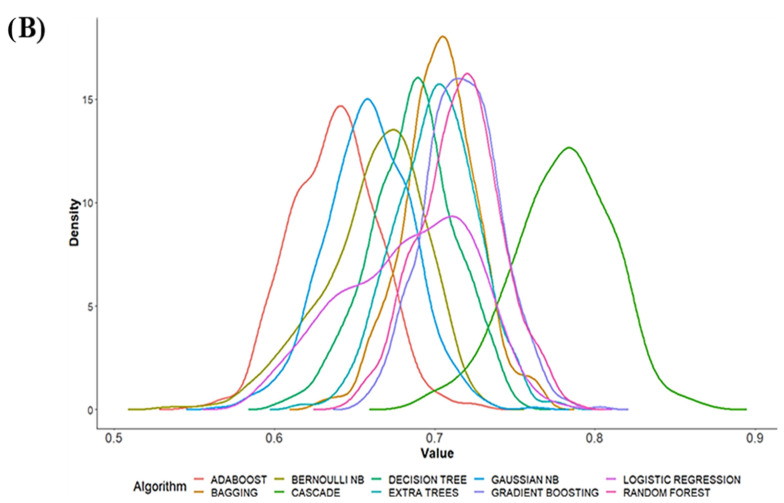
Bayesian ACC and density ACC using instances. Probability of wining for each classical machine learning technique separately and the cascade model. Panel (**A**): Results represented as Bayesian average coverage criterion. Panel (**B**): Results represented as density average coverage criterion.

Table 4 shows the rankings for each algorithm/instance. The results reveal that the cascade classifier achieved the top-ranking position in all instances, followed by the gradient boosting and random forest models. The cascade classifier achieved a statistically significantly better ranking compared to the rest of the predictive models tested. In addition, Figure 2A represents the probability of being the best method, denoted as the probability of winning, and its standard deviation for the results obtained with accuracy as the objective function. The cascade model had the highest probability of winning, with the lowest deviation compared to the rest of the models and without competing models in the same space. It was also supported by the ranking of models and represented by a density plot as a function of the accuracy obtained by the models in all their versions (Figure 2B). As shown, the cascade model concentrates most of the results around 79% accuracy, while the rest of the models presented 70% or lower (Figure 2B). All the results show that the cascade classifier is the best classifier model for predicting overweight/obesity among all the models tested.

### 3.5. Variables with the Greatest Impact on Overweight/Obesity Predictions: Interpretation of Personalized Prediction

**Figure 3:**
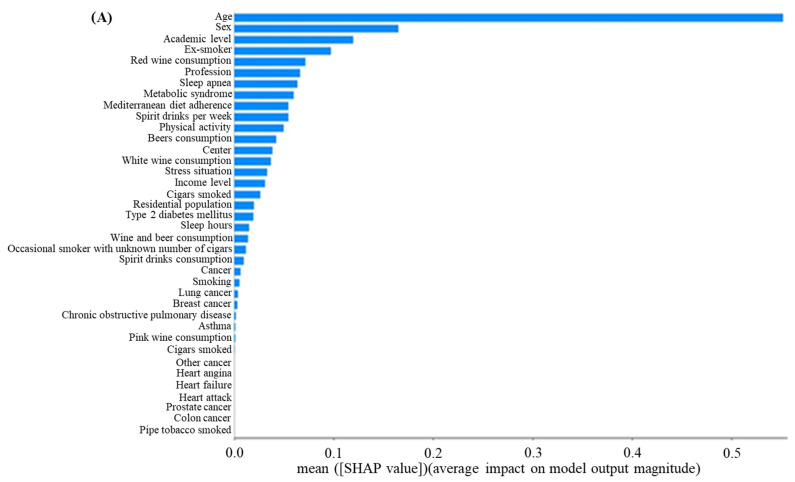

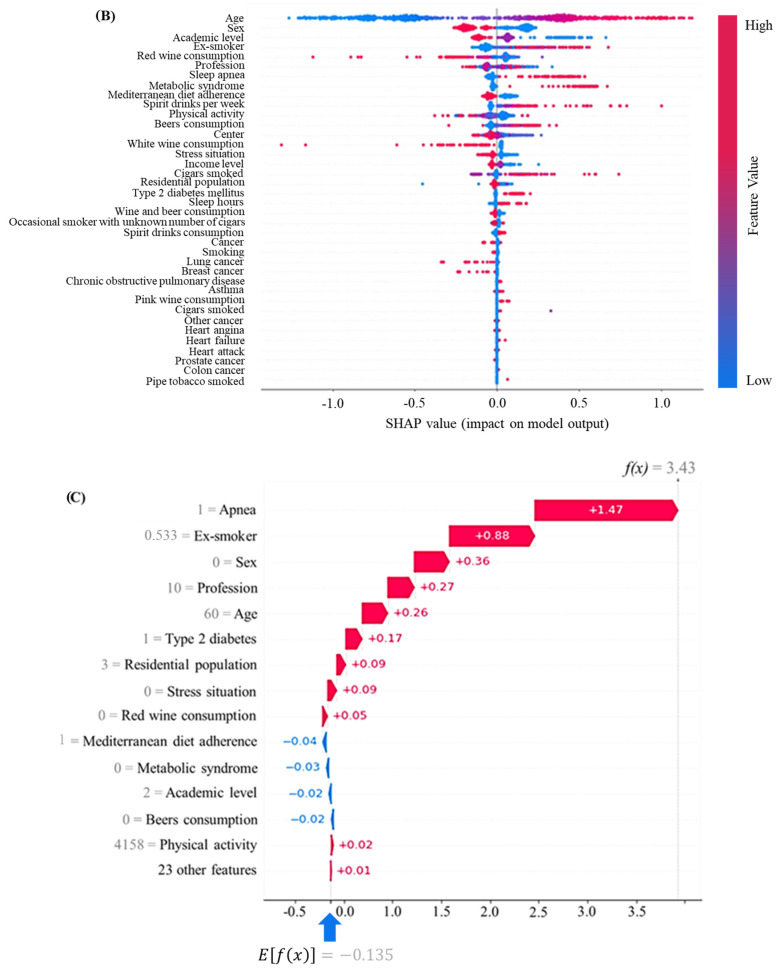
SHAP values. The model´s interpretation. Panel (**A**): Feature importance plot (after feature normalization). Panel (**B**): SHAP summary plots of the cascade flow model. Each row in the SHAP summary plot represents a feature, with the corresponding Shap values displayed along the *x*-axis. The features are ranked according to their average absolute Shap values, which represent the most important features of the model. Each point in the plot corresponds to a sample, with the color indicating the magnitude of the feature value, where red denotes larger values and blue denotes smaller values. Panel (**C**): Waterfall plot for explaining an individual´s prediction results in the validation cohort. The *y* axis shows the name of the variables and the *x* axis shows the Shap value. The red bar shows the positive contribution of the feature to the predicted value, and the blue bar shows the negative contribution of the feature to the predicted value.

Figure 3A shows the feature importance plot based on the cascade flow model. The model´s SHAP interpretation revealed that age was the variable with the greatest impact on predicting overweight/obesity (Figure 3A). Other variables, such as sex, education level, profession, and aspects related to smoking and alcohol consumption; several disorders, including apnea and metabolism syndrome; Mediterranean diet adherence; and physical activity, were important predictive factors for gaining weight, with the MDI ranging from 0.05 to 0.18.

Figure 3B represents the SHAP summary plot of the cascade flow model. Each row in the plot represents a feature, with the corresponding SHAP values displayed along the *x*-axis. The features are ranked according to their average absolute SHAP values, which represent the most important features of the model. A dot is created for each feature attribution value for the profile of each patient, and thus, one patient is allocated one dot on the line for each feature. The color indicates the magnitude of the feature value, where red denotes larger values and blue denotes smaller values. Age is a highly important feature of the model. Individuals of an older age are associated with higher red-dot values, with corresponding SHAP values greater than zero indicating a positive impact on overweight/obesity classifications. Conversely, as the feature value decreases, the SHAP value is less than zero, indicating a negative impact. In addition, the distribution of points also provides important information. The dispersion of samples in the plot for the age, sex, and education level features suggests a greater influence of these features on the model. Conversely, for the stress or earning features, most points are concentrated around SHAP = 0, indicating that these features only affect a small subset of individuals. For diseases such as sleep apnea and metabolic syndrome, as well as weekly consumption of spiritual drinks, we observed a dense cluster of instances with blue points with smaller, negative SHAP values. Instances with red points further extend toward the right, indicating that the positive impact of sleep apnea, metabolic syndrome, and spiritual drink consumption on overweight/obesity is greater than the negative impact. This suggests that patients older than 50 years are at a higher risk of gaining weight. It is important to remark that, due to the dispersion values of SHAP values for each sex, it should be interpreted that the behavior of the sex variable seems to play a bimodal role closely related to age, with the highest risk of obesity in women occurring in old age and in men in middle age. Taking all the data together, it may be considered that the profile with the highest risk of overweight/obesity would be constituted by females older than 50 years, with low educational and economic levels, ex-smokers or non-smokers, a low adherence to the Mediterranean diet, weekly beer and/or wine consumption, sedentary lifestyle, and diagnosed with chronic disorders, such as apnea or/and metabolism syndrome (Figure 3B). 

On the other hand, a typical example is provided to illustrate the interpretability of the model: a 60-year-old-male ex-smoker who had been diagnosed with sleep apnea and diabetes (Figure 3C). The arrows show the influence of each factor on prediction. The SHAP value for each characteristic is displayed as a force to increase or decrease the evaluation, and every prediction began with the base value (−0.135), which was the average SHAP value of all predictions. The blue and red arrows indicate whether the factor reduced (blue) or increased (red) the risk of overweight/obesity. The combined effects of all factors provided the final SHAP value. As is shown in Figure 3C, the SHAP value of this subject is 3.43, which is larger than the base value (−0.135), indicating a high risk of gaining weight. Among all factors, a positive (red) apnea diagnosis and being an ex-smoker, with +1.47 and +0.88, respectively, had a great contribution to the assessment of the overweight/obesity risk in the analyzed subject.

## 4. Discussion

In the present study, an interpretable innovative classifier model based on a three-stage classification model was constructed. The performed classifier, through evaluating different modifiable lifestyle variables and non-modifiable biological factors, showed a favorable predictive capability with high accuracy to classify people at risk of overweight/obesity among subjects aged 18 years or older in the community of Madrid, Spain.

Several techniques have been created with the aim of building predictive and prognostic models for different disorders, including obesity. In the last decade, machine learning algorithms have received special interest due to their promising potential in obesity research, being considered classifier models with supervised learning phases as the best option to achieve impressive high predictive accuracy [31,32,33,34,35]. Interestingly, Yi et al. analyzed the suggested superiority of deep learning for obesity predictions over traditional machine learning methods [36]. However, it is important to note that a deep learning requires much larger datasets than traditional machine learning to achieve an optimal performance. In addition, training deep learning models can be computationally intensive and requires significant resources, such as powerful processing units that are not available in healthcare or nutritional practices. On the other hand, traditional machine learning can work effectively with smaller datasets and can be less demanding in terms of computational resources. In addition, deep learning, due to its hierarchical structure and ability to learn complex features, can be less interpretable compared to traditional machine learning. This means that deep learning models can provide accurate predictions, but it is not always easy to understand how they arrived at those conclusions. On the other hand, traditional machine learning models are often more interpretable and offer a better ability to explain their results. At present, a large number of studies have presented different machine learning approaches to predict obesity [37]. However, these studies are very heterogeneous with respect to machine learning techniques, risk factors, and populations tested. This heterogeneity makes it difficult to perform a comparison among them. For example, Singh and Tawfik analyzed numerous multivariate regression algorithms on a dataset obtained from a millennium cohort and acquired over 93.4% accuracy to predict teenage BMIs [38]. Uçar et al. estimated individual percentages of body fat using hybrid machine learning algorithms, such as the support vector machine regression model and decision tree regression, using 13 anthropometric measurements [39], while Zheng et al. used binary logistic regression, improved decision tree, and even artificial neural network models on nine health-related behaviors to predict obesity in high-school students, obtaining accuracy levels ranging between 80.23 and 84.22%, depending on the technique used [40]. However, most studies seem to have in common the use of different predictive techniques independently, without analyzing combined models, and with a limited number of inputs in the dataset. In the present study, a classifier model was developed based on the combination of three supervised machine learning algorithms using 38 different inputs associated with lifestyle, sociodemographic, and health status variables, which allowed for an accuracy of 79% for predicting overweight/obesity. Interestingly, the proposed classifier model achieved the highest value of accuracy when it was compared to nine classical machine learning methods separately, whose accuracy results ranged from 63 to 73%. In addition, it was also demonstrated that there were significant differences in metric variables, such as precision and recall, between classical algorithms separately and the combined predictive model, showing the higher predictive ability of the combined model. Our findings suggest that the successful implementation and evaluation of the combined model might offer valuable insights into the development of more robust and accurate machine learning systems for addressing overweight/obesity-related challenges. In this line of evidence, recent studies also reported that combining different machine learning algorithms succeeded in predicting obesity with higher accuracy values than individual models separately [37]. Surprisingly, other studies have obtained exceptionally high accuracy values, ranging from 89.0% to 97.2% [41,42]. However, it is important to remark that they may have achieved such good results due to the inclusion of height and weight as inputs for their combined models [41,42]. Even other studies include a very limited number of inputs, which also included height and weight. In this regard, Jindal et al. employed an ensemble machine learning approach for predicting obesity based on four main determinants (age, height, weight, and BMI), obtaining average predicted values very close to 90% [43]. In contrast, weight and height variables were not included as inputs in the training process of the predictive model proposed in the present work, since both variables define the subject´s body mass index and, therefore, the predictive model would recognize these cases. The non-inclusion of weight and height affects the accuracy; however, it supports the higher robustness of the predictive model.

In general, the machine learning approach has become a powerful tool that leads to a better understanding of multifactorial disorders, such as obesity [44]. In this regard, ma-chine learning has been shown to have the ability to identify factors with a higher impact on obesity and even the most significant interactions among those risk factors for predicting obesity [45]. In accordance with other studies, our results reveal that age and sex have the greatest impact on overweight/obesity [7,46]. It is widely known that aging is closely associated with an increase in abdominal white adipose tissue and fat deposition in skeletal muscle [47,48,49]. However, according to the predictive model proposed, the behavior of the sex variable seems to play a bimodal role closely related to age, with the highest risk of obesity in women occurring in old age and in men in middle age. At least, in part, this differential behavior may be explained by biological factors or hormonal changes associated with different stages of life. Indeed, in females, menopause directly affects fat distribution and deposition, increasing the risk of overweight and obesity [50,51]. It suggests that the cascade flow algorithm may have the ability to present the physiological aspects related to sex and age and is closely involved in weight gain.

The present study also determined a considerable impact on two variables related to health status, reporting that sleep apnea and metabolic syndrome diagnoses may act as obesity predictors. Similar findings have been previously described in other studies [52,53]. However, honestly, it would be more plausible to consider that obesity is the main cause of sleep apnea and metabolic syndrome. However, it is important to point out that other aspects of the above-mentioned disorders may promote higher obesity. In this regard, one of the underlying mechanisms of metabolic syndrome is insulin resistance, which is widely accepted to promote hyperglycemia and higher abdominal adiposity [54,55]. On the other hand, alterations in the sleep cycle due to sleep apnea induce important neuroendocrine and metabolic modifications strongly associated with obesity [56,57,58,59]. Interestingly, in the present study, it has been also identified with sleep less than 8 h as a predictor for weight gain.

A large number of studies have established a close relationship between lifestyle habits and overweight/obesity, paying special attention to dietary patterns and physical exercise [60,61]. Consequently, our results demonstrate that nutritional habits and physical activity as well as toxic habits have a great impact on overweight/obesity classification. In this regard, it was observed that an adherence to the Mediterranean diet and vigorous or medium physical activity were negative predictors for overweight/obesity. These findings are supported by previous studies using conventional statistical analysis techniques and machine learning techniques [62,63]. On the other hand, the proposed model in the present study revealed that toxic habits also seem to play an important role in predicting overweight/obesity. In this regard, there was a directly proportional relationship between the probability of becoming overweight and being an ex-smoker. Interestingly, epidemiologic studies have reported that, on average, smokers tend to weigh less than non-smokers. The majority of quitters gain about 3–9 kg within 8 years of quitting, and between 10% and 13% of quitters gain at least 11 kg [64,65,66,67]. With respect to alcoholic drinks consumption, several studies have reported that alcohol consumption does not necessarily lead to weight gain [68,69], a reduction in the risk of overweight/obesity being reported among moderate alcohol consumers compared to non-drinkers, showing that the beneficial effect of drinking on obesity is present when alcohol is consumed in low–moderate amounts on a regular basis [69,70]. Consistent with these findings, the moderate and low consumption of beer and/or wine were used by the cascade flow model as negative predictors for developing overweight/obesity.

Additionally, in accordance with previous studies, several sociodemographic factors, such as academic level, profession, and even economic status, have an importance effect on the risk of being overweight or obese [71,72]. However, the effects of sociodemographic factors on overweight and obesity risk are paradoxical, with controversial results in the scientific literature [73,74]. At least, in part, this may be due to several sociodemographic variables that seem to be related to and even conditioned by other predictive variables of weight gain. In this regard, type of profession exerts an important influence on the level of physical activity [75], which is one of the most powerful predictors of obesity, as discussed above. It highlights the need to create predictive models that allow us to assess not only the impact of each variable on weight gain, but also the effect of the relationship among different variables on overweight/obesity risk. Accordingly, different authors have concluded that classifier models using machine learning techniques could be used to develop individualized predictions based on specific individual features and interactions among subject features, while logistic regression models should be only applied at the population level [76,77]. In the present study, the interpretation of the SHAP value was used to combine the predictive model to help better understand the decision-making process. The SHAP value assesses the significance of the output by considering all possible feature combinations and provides consistent and locally precise attribute values for each feature in the prediction model. In summary, considering the key risk factors, the model can intuitively explain to clinicians which specific characteristics of patients predispose them to a higher or lower risk of developing overweight/obesity. Our interpretable classifier model has the potential to find the most specific features involved in obesity for each subject and, therefore, to personalize prevention strategies, rendering it a valuable tool in clinical practice.

The results obtained with our predictive model are robust, based on the analysis of the modifiable and biological non-modifiable factors closely associated with overweight/obesity in a large sample size and the high quality of the dataset and effect size. However, it also shows limitations. One of them is that relationship between some predictors and obesity must be interpreted as “reverse causality”. In this regard, sedentarism has been identified as a relevant predictor of obesity, however it is not easy to establish if obesity is caused by sedentarism or if obesity makes it impossible to practice physical activity. This fact may be considered as a common limiting factor to approaches by predictive algorithms. In addition, several factors that might influence obesity, such as genetic profile, cultural influences, or psychological aspects, were not included in our analysis. An important advantage of machine learning techniques is that the inclusion of new factors would not discard the validity of the predictive model, but it would also allow obtaining higher precision in the prediction model.

## 5. Conclusions

- Three-stage classification model based on a combination of machine learning techniques showed a significant improvement in accuracy to predict risk of overweight/obesity than machine learning techniques separately.
- The predictive model created and SHAP technique had the ability to show those individualized modifiable factors with significant impacts on weight gain. This offers a transparent explanation of personalized risk prediction, enabling health professionals to gain an intuitive understanding of the impact of key features in the model.
- More studies are needed to further improve the quality of predictions, exploring the effect of other factors not included in the dataset. The validation of the results might help to optimize the designs of health policies and programs to decrease obesity incidence/prevalence and, in turn, reduce the severity as well as the cost of treating obesity and obesity-related conditions, which eventually could improve the health and well-being of the population.

## Figures and Tables

**Table 1:** Cross-validation results for the study population.

Model	Accuracy	Std
Bagging	0.72	0.10
Logistic Regression	0.70	0.13
Gradient Boosting	0.71	0.10
Extra Trees	0.70	0.10
Random Forest	0.69	0.08
Gaussian Nb	0.67	0.12
Bernoulli Nb	0.67	0.10
Decision Tree	0.64	0.05
AdaBoost	0.63	0.05

Std: Standard deviation.

**Table 2:** Results of metric variables for the cascade classifier model.

Metric Variables	Categories	Instance 38 (N = 295)	Instance 37a (N = 295)	Instance 37c (N = 295)
*Correct classification rate*		0.81	0.79	0.80
*Subjects classified*		193	151	175
*Subjects unclassified*		102	144	120
*Variable number*		38	37	37
Precision	Normal weight	0.85	0.86	0.78
	Overweight/obesity	0.78	0.76	0.82
	Macro avg	0.81	0.81	0.80
	Weighted avg	0.81	0.81	0.80
Recall	Normal weight	0.75	0.68	0.79
	Overweight/obesity	0.87	0.90	0.81
	Macro avg	0.81	0.79	0.80
	Weighted avg	0.81	0.79	0.80
F1-score	Normal weight	0.79	0.76	0.79
	Overweight/obesity	0.82	0.82	0.81
	Macro avg	0.81	0.79	0.80
	Weighted avg	0.81	0.79	0.80

Instance 38: 38 variables; instance 37a: 37 variables without age; instance 37c: 37 variables without center.

**Table 3:** Results after 100 runs of each algorithm for each of the instances.

Algorithm	Instance	Accuracy				Precision		Recall	
		Best	Worst	Mean	Std	Normal Weight	Overweight/Obesity	Normal Weight	Overweight/Obesity
AdaBoost	38	0.7119	0.5661	0.6297	0.0255	0.7312	0.6889	0.7358	0.6838
AdaBoost	37a	0.7017	0.5797	0.6448	0.0271	0.7386	0.6620	0.7019	0.7015
AdaBoost	37c	0.6915	0.5864	0.6406	0.0208	0.6795	0.7050	0.7211	0.6622
Bagging	38	0.7695	0.6576	0.7091	0.0251	0.7925	0.7426	0.7826	0.7537
Bagging	37a	0.7356	0.6271	0.6921	0.0221	0.7635	0.7075	0.7244	0.7482
Bagging	37c	0.7627	0.6542	0.7141	0.0212	0.7484	0.7786	0.7891	0.7365
Bernoulli NB	38	0.7390	0.5932	0.6718	0.0275	0.7386	0.7395	0.8075	0.6567
Bernoulli NB	37a	0.7356	0.6271	0.6745	0.0221	0.7222	0.7565	0.8228	0.6350
Bernoulli NB	37c	0.7763	0.6271	0.6760	0.0266	0.7596	0.8036	0.8634	0.6716
Decision Tree	38	0.7492	0.6271	0.6907	0.0235	0.7322	0.7768	0.8428	0.6397
Decision Tree	37a	0.7661	0.6136	0.6823	0.0266	0.7486	0.7917	0.8397	0.6835
Decision Tree	37c	0.7458	0.6237	0.6882	0.0252	0.7485	0.7422	0.7911	0.6934
Extra Trees	38	0.7695	0.6576	0.7094	0.0217	0.7484	0.7941	0.8095	0.7297
Extra Trees	37a	0.7593	0.6305	0.6889	0.0251	0.7831	0.7287	0.7879	0.7231
Extra Trees	37c	0.7627	0.6508	0.7101	0.0249	0.7419	0.7857	0.7931	0.7333
Gradient Boosting	38	0.7763	0.6576	0.7256	0.0206	0.7701	0.7851	0.8375	0.7037
Gradient Boosting	37a	0.7559	0.6576	0.7094	0.0226	0.7803	0.7213	0.7988	0.6984
Gradient Boosting	37c	0.7864	0.6576	0.7284	0.0222	0.7747	0.8053	0.8650	0.6894
Gaussian NB	38	0.7085	0.6102	0.6590	0.0221	0.6814	0.7971	0.9167	0.4331
Gaussian NB	37a	0.6949	0.6034	0.6517	0.0214	0.6524	0.8000	0.8896	0.4823
Gaussian NB	37c	0.7390	0.5932	0.6584	0.0235	0.7110	0.8182	0.9172	0.5000
Logistic Regression	38	0.7898	0.6610	0.7115	0.0234	0.8042	0.7763	0.7718	0.8082
Logistic Regression	37a	0.7017	0.5831	0.6446	0.0241	0.6871	0.7162	0.7063	0.6974
Logistic Regression	37c	0.7729	0.6644	0.7144	0.0214	0.7425	0.8125	0.8378	0.7075
Random Forest	38	0.7797	0.6814	0.7183	0.0190	0.7546	0.8106	0.8311	0.7279
Random Forest	37a	0.7458	0.6407	0.6999	0.0220	0.7284	0.7669	0.7919	0.6986
Random Forest	37c	0.7763	0.6644	0.7255	0.0236	0.7582	0.7958	0.8000	0.7533
Cascade Classifier	38	0.8678	0.7320	0.7926	0.0283	0.8442	0.8866	0.8553	0.8776
Cascade Classifier	37a	0.8395	0.6875	0.7704	0.0330	0.8923	0.8041	0.7532	0.9176
Cascade Classifier	37c	0.8432	0.7287	0.7968	0.0234	0.8295	0.8557	0.8391	0.8469

Std: Standard deviation.

**Table 4:** Ranks for each algorithm/instance.

Instance	AdaBoost	Bagging	Bernoulli NB	Decision Tree	Extra Trees	GB	Gaussian NB	LR	RF	Cascade
38	10	5.5	8	7	5.5	2	9	4	3	1
37a	9.5	4	7	6	5	2	8	9.5	3	1
37c	10	4.5	8	7	6	2	9	4.5	3	1
Average	9.8	4.7	7.7	6.7	5.5	2	8.7	6	3	1
Std	0.3	0.7	0.6	0.6	0.5	0	0.6	3.0	0	0

GB: gradient boosting; LR: logistic regression; RF: random forest; Std: standard deviation.

## Data Availability

The data that support the findings of this study are available on reasonable request from the corresponding authors.

## Supplementary Materials

The following supporting information can be downloaded at: https://www.mdpi.com/article/10.3390/jpm14080816/s1, Supplementary Table S1: Definition of variables and categories and description of study population. Supplementary Table S2: Results after 100 runs of each algorithm for instance 36 (36 variables without age or recruitment center).

## References

[1] Consumer Affairs (2017). Ministry of Health and Social Welfare. National Health Survey. Spain. https://www.sanidad.gob.es/estadEstudios/estadisticas/encuestaNacional/encuestaNac2017/ENSE2017_notatecnica.pdf.

[2] Fruh S.M. (2017). Obesity: Risk factors, complications, and strategies for sustainable long-term weight management. J. Am. Assoc. Nurse Pract..

[3] WHO (2024). Obesity and Overweight. https://www.who.int/news-room/fact-sheets/detail/obesity-and-overweight.

[4] Telleria-Aramburu N., Arroyo-Izaga M. (2022). Risk factors of overweight/obesity-related lifestyles in university students: Results from the EHU12/24 study. Br. J. Nutr..

[5] Cheadle A., Atiedu A., Rauzon S., Schwartz P.M., Keene L., Davoudi M., Spring R., Molina M., Lee L., Boyle K. (2018). A Community-Level Initiative to Prevent Obesity: Results from Kaiser Permanente’s Healthy Eating Active Living Zones Initiative in California. Am. J. Prev. Med..

[6] Narciso J., Silva A.J., Rodrigues V., Monteiro M.J., Almeida A., Saavedra R., Costa A.M. (2019). Behavioral, contextual and biological factors associated with obesity during adolescence: A systematic review. PLoS ONE.

[7] Chatterjee A., Gerdes M.W., Martinez S.G. (2020). Identification of Risk Factors Associated with Obesity and Overweight—A Machine Learning Overview. Sensors.

[8] Börnhorst C., Russo P., Veidebaum T., Tornaritis M., Molnár D., Lissner L., Mårild S., De Henauw S., Moreno L.A., Floegel A. (2020). The role of lifestyle and non-modifiable risk factors in the development of metabolic disturbances from childhood to adolescence. Int. J. Obes..

[9] Hruby A., Hu F.B. (2015). The Epidemiology of Obesity: A Big Picture. Pharmacoeconomics.

[10] Battineni G., Sagaro G.G., Chinatalapudi N., Amenta F. (2020). Applications of Machine Learning Predictive Models in the Chronic Disease Diagnosis. J. Pers. Med..

[11] Dick S. (2019). Artificial intelligence. Harv. Data Sci. Rev..

[12] Scheinker D., Valencia A., Rodriguez F. (2019). Identification of Factors Associated with Variation in US County-Level Obesity Prevalence Rates Using Epidemiologic vs. Machine Learning Models. JAMA Netw. Open.

[13] DeGregory K.W., Kuiper P., DeSilvio T., Pleuss J.D., Miller R., Roginski J.W., Fisher C.B., Harness D., Viswanath S., Heymsfield S.B. (2018). A review of machine learning in obesity. Obes. Rev..

[14] Golino H.F., Amaral L.S.D.B., Duarte S.F.P., Gomes C.M.A., Soares T.D.J., Reis L.A.D., Santos J. (2014). Predicting increased blood pres-sure using machine learning. J. Obes..

[15] Pleuss J.D., Talty K., Morse S., Kuiper P., Scioletti M., Heymsfield S.B., Thomas D.M. (2019). A machine learning approach relating 3D body scans to body composition in humans. Eur. J. Clin. Nutr..

[16] Maharana A., Nsoesie E.O. (2018). Use of deep learning to examine the association of the built environment with prevalence of neighborhood adult obesity. JAMA Netw. Open.

[17] Pouladzadeh P., Kuhad P., Peddi S.V.B., Yassine A., Shirmohammadi S. Food calorie measurement using deep learning neural network. Proceedings of the 2016 IEEE International Instrumentation and Measurement Technology Conference.

[18] De-La-Hoz-Correa E., Mendoza Palechor E., De-La-Hoz-Manotas E., Morales Ortega A., Sánchez Hernández R., Adriana B. (2019). Obesity level estimation software based on decision trees. J. Comput. Sci..

[19] Singh B., Tawfik H. Machine Learning Approach for the Early Prediction of the Risk of Overweight and Obesity in Young People. Proceedings of the Computational Science—ICCS 2020: 20th International Conference.

[20] Breiman L. (1994). Bagging predictors. J. Time Ser. Anal..

[21] Natekin A., Knoll A. (2013). Gradient boosting machines, a tutorial. Front. Neurorobotics.

[22] Breiman L. (2001). Random forests. Mach. Learn..

[23] Monaghan T.F., Rahman S.N., Agudelo C.W., Wein A.J., Lazar J.M., Everaert K., Dmochowski R.R. (2021). Foundational Statistical Principles in Medical Research: Sensitivity, Specificity, Positive Predictive Value, and Negative Predictive Value. Medicina.

[24] Friedman M. (1937). The use of ranks to avoid the assumption of normality implicit in the analysis of variance. J. Am. Stat. Assoc..

[25] Friedman M. (1940). A Comparison of Alternative Tests of Significance for the Problem of m Rankings. Ann. Math. Stat..

[26] Derrac J., García S., Molina D., Herrera F. (2011). A practical tutorial on the use of nonparametric statistical tests as a methodology for comparing evolutionary and swarm intelligence algorithms. Swarm Evol. Comput..

[27] Futagami K., Fukazawa Y., Kapoor N., Kito T. (2021). Pairwise acquisition prediction with SHAP value interpretation. J. Financ. Data Sci..

[28] Mangalathu S., Hwang S.H., Jeon J.S. (2020). Failure mode and effects analysis of RC members based on machine-learning-based Shapley Additive Explanations (SHAP) approach. Eng. Struct..

[29] Zeng W., Davoodi A., Topaloglu R.O. Explainable DRC Hotspot Prediction with Random Forest and SHAP Tree Explainer. Proceedings of the 2020 Design, Automation & Test in Europe Conference & Exhibition (DATE).

[30] Lundberg S.M., Lee S.I. (2017). Unified approach to interpreting model predictions. Adv. Neural Inf. Process. Syst..

[31] Zou Q., Qu K., Luo Y., Yin D., Ju Y., Tang H. (2018). Predicting Diabetes Mellitus with Machine Learning Techniques. Front. Genet..

[32] Acharjee A., Ament Z., West J.A., Stanley E., Griffin J.L. (2016). Integration of metabolomics, lipidomics and clinical data using a machine learning method. BMC Bioinform..

[33] Dugan T.M., Mukhopadhyay S., Carroll A., Downs S. (2015). Machine Learning Techniques for Prediction of Early Childhood Obesity. Appl. Clin. Inform..

[34] Ellis K., Kerr J., Godbole S., Staudenmayer J., Lanckriet G. (2016). Hip and Wrist Accelerometer Algorithms for Free-Living Behavior Classification. Med. Sci. Sports Exerc..

[35] Triantafyllidis A., Polychronidou E., Alexiadis A., Rocha C.L., Oliveira D.N., da Silva A.S., Freire A.L., Macedo C., Sousa I.F., Werbet E. (2020). Computerized decision support and machine learning applications for the prevention and treatment of childhood obesity: A systematic review of the literature. Artif. Intell. Med..

[36] Yi X., He Y., Gao S., Li M. (2024). A review of the application of deep learning in obesity: From early prediction aid to advanced management assistance. Diabetes Metab. Syndr. Clin. Res. Rev..

[37] Safaei M., Sundararajan E.A., Driss M., Boulila W., Shapi’i A. (2021). A systematic literature review on obesity: Understanding the causes & consequences of obesity and reviewing various machine learning approaches used to predict obesity. Comput. Biol. Med..

[38] Singh B., Tawfik H. A Machine Learning Approach for Predicting Weight Gain Risks in Young Adults. Proceedings of the 2019 10th International Conference on Dependable Systems, Services and Technologies (DESSERT).

[39] Uçar M.K., Uçar Z., Köksal F., Daldal N. (2021). Estimation of body fat percentage using hybrid machine learning algorithms. Measurement.

[40] Zheng Z., Ruggiero K. Using machine learning to predict obesity in high school students. Proceedings of the 2017 IEEE International Conference on Bioinformatics and Biomedicine (BIBM).

[41] Solomon D.D., Khan S., Garg S., Gupta G., Almjally A., Alabduallah B.I., Alsagri H.S., Ibrahim M.M., Abdallah A.M.A. (2023). Hybrid Majority Voting: Prediction and Classification Model for Obesity. Diagnostics.

[42] Taghiyev A., Altun A.A., Caglar S.A. (2020). Hybrid Approach Based on Machine Learning to Identify the Causes of Obesity. J. Control Eng. Appl. Inform..

[43] Jindal K., Baliyan N., Rana P.S. (2018). Obesity prediction using ensemble machine learning approaches. Recent Findings in Intelligent Computing Techniques.

[44] Ngiam K.Y., Khor I.W. (2019). Big data and machine learning algorithms for health-care delivery. Lancet Oncol..

[45] Thamrin S.A., Arsyad D.S., Kuswanto H., Lawi A., Nasir S. (2021). Predicting Obesity in Adults Using Machine Learning Techniques: An Analysis of Indonesian Basic Health Research 2018. Front. Nutr..

[46] Lin W., Shi S., Huang H., Wen J., Chen G. (2023). Predicting risk of obesity in overweight adults using interpretable machine learning algorithms. Front. Endocrinol..

[47] Mancuso P., Bouchard B. (2019). The Impact of Aging on Adipose Function and Adipokine Synthesis. Front. Endocrinol..

[48] Wang X., Xu M., Li Y. (2022). Adipose Tissue Aging and Metabolic Disorder, and the Impact of Nutritional Interventions. Nutrients.

[49] Conte M., Martucci M., Sandri M., Franceschi C., Salvioli S. (2019). The Dual Role of the Pervasive “Fattish” Tissue Remodeling with Age. Front. Endocrinol..

[50] Davis S.R., Castelo-Branco C., Chedraui P., Lumsden M.A., Nappi R.E., Shah D., Villaseca P., Writing Group of the International Menopause Society for World Menopause Day 2012 (2012). Understanding weight gain at menopause. Climacteric.

[51] Milewicz A., Tworowska U., Demissie M. (2001). Menopausal obesity–myth or fact?. Climacteric.

[52] Kostoglou-Athanassiou I., Athanassiou P. (2008). Metabolic syndrome and sleep apnea. Hippokratia.

[53] Lam J.C., Mak J.C., Ip M.S. (2012). Obesity, obstructive sleep apnea and metabolic syndrome. Respirology.

[54] Zhao X., An X., Yang C., Sun W., Ji H., Lian F. (2023). The crucial role and mechanism of insulin resistance in metabolic disease. Front. Endocrinol..

[55] Li M., Chi X., Wang Y., Setrerrahmane S., Xie W., Xu H. (2022). Trends in insulin resistance: Insights into mechanisms and therapeutic strategy. Signal Transduct. Target. Ther..

[56] Patel S.R., Hu F.B. (2008). Short sleep duration and weight gain: A systematic review. Obesity.

[57] Leproult R., Van Cauter E. (2010). Role of sleep and sleep loss in hormonal release and metabolism. Endocr. Dev..

[58] Cappuccio F.P., Miller M.A., Cappuccio F.P., Miller M.A., Lockley S.W. (2010). The epidemiology of sleep and cardiovascular risk and disease. Sleep, Health and Society: From Aetiology to Public Health.

[59] Cappuccio F.P., Miller M.A., Lockley S.W., Rajaratnam S.M.W. (2018). Sleep, Health, and Society: From Aetiology to Public Health.

[60] Kerkadi A., Sadig A.H., Bawadi H., Al Thani A.A.M., Al Chetachi W., Akram H., Al-Hazzaa H.M., Musaiger A.O. (2019). The Relationship between Lifestyle Factors and Obesity Indices among Adolescents in Qatar. Int. J. Environ. Res. Public Health.

[61] Petrella E., Malavolti M., Bertarini V., Pignatti L., Neri I., Battistini N.C., Facchinett F. (2014). Gestational weight gain in overweight and obese women enrolled in a healthy lifestyle and eating habits program. J. Matern.-Fetal Neonatal Med..

[62] Rosi A., Giopp F., Milioli G., Melegari G., Goldoni M., Parrino L., Scazzina F. (2020). Weight Status, Adherence to the Mediterranean Diet, Physical Activity Level, and Sleep Behavior of Italian Junior High School Adolescents. Nutrients.

[63] Cheng X., Lin S.Y., Liu J., Liu S., Zhang J., Nie P., Fuemmeler B.F., Wang Y., Xue H. (2021). Does Physical Activity Predict Obesity-A Machine Learning and Statistical Method-Based Analysis. Int. J. Environ. Res. Public Health.

[64] Williamson D.F., Madans J., Anda R.F., Kleinman J.C., Giovino G.A., Byers T. (1991). Smoking cessation and severity of weight gain in a national cohort. N. Engl. J. Med..

[65] Pisinger C., Jorgensen T. (2007). Weight concerns and smoking in a general population: The Inter99 study. Prev. Med..

[66] Lycett D., Munafò M., Johnstone E., Murphy M., Aveyard P. (2011). Associations between weight change over 8 years and baseline body mass index in a cohort of continuing and quitting smokers. Addiction.

[67] O’Hara P., Connett J.E., Lee W.W., Nides M., Murray R., Wise R. (1998). Early and late weight gain following smoking cessation in the Lung Health Study. Am. J. Epidemiol..

[68] Kase C.A., Piers A.D., Schaumberg K., Forman E.M., Butryn M.L. (2016). The relationship of alcohol use to weight loss in the context of behavioral weight loss treatment. Appetite.

[69] Tolstrup J.S., Heitmann B.L., Tjønneland A.M., Overvad O.K., Sørensen T.I., Grønbaek M.N. (2005). The relation between drinking pattern and body mass index and waist and hip circumference. Int. J. Obes..

[70] Arif A.A., Rohrer J.E. (2005). Patterns of alcohol drinking and its association with obesity: Data from the Third National Health and Nutrition Examination Survey, 1988–1994. BMC Public Health.

[71] Wang K., Wu C., Yao Y., Zhang S., Xie Y., Shi K., Yuan Z. (2022). Association between socio-economic factors and the risk of overweight and obesity among Chinese adults: A retrospective cross-sectional study from the China Health and Nutrition Survey. Glob. Health Res. Policy.

[72] Rummo P.E., Feldman J.M., Lopez P., Lee D., Thorpe L.E., Elbel B. (2020). Impact of Changes in the Food, Built, and Socioeconomic Environment on BMI in US Counties, BRFSS 2003–2012. Obesity.

[73] Ohlsson B., Manjer J. (2020). Sociodemographic and Lifestyle Factors in relation to Overweight Defined by BMI and “Normal-Weight Obesity”. J. Obes..

[74] Pou S.A., Diaz M.D.P., Velázquez G.A., Aballay L.R. (2022). Sociodemographic disparities and contextual factors in obesity: Updated evidence from a National Survey of Risk Factors for Chronic Diseases. Public Health Nutr..

[75] Van Domelen D.R., Koster A., Caserotti P., Brychta R.J., Chen K.Y., McClain J.J., Troiano R.P., Berrigan D., Harris T.B. (2011). Employment and physical activity in the U.S. Am. J. Prev. Med..

[76] Hosmer D.W., Lemeshow S., Sturdivant R.X. (2013). Applied Logistic Regression.

[77] Khalaf M., Hussain A.J., Keight R., Al-Jumeily D., Fergus P., Keenan R., Tso P. (2017). Machine learning approaches to the application of disease modifying therapy for sickle cell using classification models. Neurocomputing.

