# Supplementary material for: Combination of Machine Learning Techniques to Predict Overweight/Obesity in Adults
Source: J Pers Med. 2024 Jul 31;14(8):816. doi: 10.3390/jpm14080816 (PMC11355742; doi:10.3390/jpm14080816)
Supplement: Supplementary file 1 [file jpm-14-00816-s001.zip › jpm-3103140-supplementary.pdf]

Supplementary Table S1. Description of study population.

VARIABLES	CATEGORIES	% (Frequency) N= 1179
1. Sex	1. Male	48.3 (569)
	2. Female	51.7 (610)
2. Age		41.21 ± 0.58
3. Center	1. University	44.4 (523)
	2. Hospital center	55.6 (656)
4. Residential population	1. >50.000 people	71 (837)
	2. 50.000 - 20.000 people	11.8 (139)
	3. 20.000 - 2.500 people	13 (153)
	4. < 2.500 people	4.2 (50)
5. Academic level	1. Without studies	1.2 (14)
	2. Basic (complete primary education)	9.6 (113)
	3. Secondary (complete secondary education)	48.7 (574)
	4. University (university studies or equivalent)	40.5 (478)
6. Income level	1. Low (<1.000 €)	9.3 (110)
	2. Medium (1.000 – 2.000 €)	43.9 (518)
	3. High (>2.000 €)	46.7 (551)
7. Profession	1. Directors and managers	4.2 (49)
	2. Technical and scientific intellectual professionals	19.5 (230)
	3. Technical support professionals	5.9 (69)
	4. Accounting, administrative and other office employees	8.1 (96)
	5. Catering services workers, protection and sales services	5.6 (66)
	6. Craftsmen and skilled workers in the manufacturing and construction industries	1.5 (18)
	7. Facility and machinery operators, assemblers	1.7 (20)
	8. Elementary occupations	3.9 (46)
	9. Student	32.1 (378)
	10. Unemployed	6.6 (78)
	11. Military occupations	0.2 (2)
	12. Athlete	1.8 (21)
	13. Qualified workers in agricultural, livestock, forestry and fishing sectors	0.3 (3)
	14. Retired	8.7 (103)
8. Stress situation	1. Yes	44.4 (524)
	2. No	55.6 (655)
9. Sleep hours	1. Less than 8 hours (<8h)	79.9 (942)
	2. More than 8 hours (≥8h)	20.1 (237)
10. Spirit drinks consumption	1. Yes	28.4 (335)
	2. No	71.6 (844)
11. Spirit drinks per week (Spirit Drinks/Week)		0.76 ± 0.06
12. Wine and beer consumption	1. Yes	54.5 (642)
	2. No	45.5 (537)

13. Beers consumption (Beers/Week*)		1.74 ± 0.10
14. Red wine consumption (Red Wine/Week*)		0.79 ± 0.06
15. White wine consumption (White Wine/Week*)		0.18 ± 0.03
16. Pink wine consumption (Pink Wine/Week*)		0.03 ± 0.01
17. Smoking	1. Yes	19.1 (225)
	2. No	80.9 (954)
18. Cigars smoked (Cigars/day*)		1.57 ± 0.12
19. Pipe tobacco smoked (Pipe tobacco/day*)		0.009 ± 0.005
20. Cigars smoked (Cigars/day*)		0.006 ± 0.002
21. Ex-smoker (years since quitting*)		3.25 ± 0.23
22. Occasional smoker with unknown number of cigars	1. Yes	41.8 (493)
	2. No	58.2 (686)
23. Cancer	1. Yes	7.3 (86)
	2. No	92.7 (1093)
If yes	24. Breast	1.3 (15)
	25. Colon	0.6 (7)
	26. Prostate	0.5 (6)
	27. Lung	2 (23)
	28. Other	3.1 (36)
29. Heart attack	1. Yes	2.3 (27)
	2. No	97.7 (1152)
30. Heart angina	1. Yes	1.9 (22)
	2. No	98.1 (1157)
31. Heart failure	1. Yes	1.4 (17)
	2. No	98.6 (1162)
32. Type 2 diabetes mellitus	1. Yes	9.7 (114)
	2. No	90.3 (1065)
33. Metabolic syndrome	1. Yes	7.3 (86)
	2. No	92.7 (1093)
34. Sleep apnea	1. Yes	8.7 (103)
	2. No	91.3 (1076)
35. Asthma	1. Yes	10.3 (122)
	2. No	89.7 (1057)
36. Chronic Obstructive Pulmonary Diseases	1. Yes	3.6 (42)
	2. No	96.4 (1137)
37. Mediterranean diet adherence	1. High (≥9 points at MEDAS test)	59.9 (706)
	2. Low (<9 point at MEDAS test)	40.1 (473)
38. Physical activity	1. Vigorous (at least 1500 – 3000 MET based on IPAQ test)	51.8 (611)
	2. Moderate (at least 600 MET based on IPAQ test)	22.5 (265)
	3. Light physical activity (<600 MET based on IPAQ test)	25.7 (303)
*Overweight/obesity?	1. No. Subjects with BMI ≤ 24.99 kg/m^2 (normal-weight group)	51.9 (612)
	2. Yes. Subjects with BMI >25 kg/m^2 (overweight/obesity group)	48.1 (567)

Abbreviations: MEDAS: Mediterranean adherence survey; IPAQ: International physical activity questionnaire; MET: Metabolic equivalent of task; BMI: Body mass index. *Overweight/obesity variable was used as objective variable, it was not used as input to train the predictive model based on cascade classifier flow.

Supplementary Table S2. Results after 100 runs of each algorithm for instance 36 (36 variables without age and recruitment center).

Algorithm	Best	Worse	Mean	Std	Precision 0	Precision 1	Recall 0	Recall 1
Adaboost	0.7085	0.5661	0.6445	0.0243	0.7117	0.7045	0.7484	0.6643
Bagging	0.7424	0.6237	0.6928	0.0222	0.7325	0.7536	0.7718	0.7123
Bernoulli NB	0.7220	0.6237	0.6743	0.0255	0.6919	0.7642	0.8041	0.6395
Decision Tree	0.7322	0.6271	0.6764	0.0230	0.7349	0.7287	0.7771	0.6812
Extra Trees	0.7390	0.6305	0.6833	0.0211	0.7091	0.7769	0.8014	0.6779
Gradient Boosting	0.7831	0.6508	0.7071	0.0230	0.7844	0.7812	0.8239	0.7353
Gaussian NB	0.7186	0.6034	0.6573	0.0256	0.6968	0.7838	0.9059	0.4640
Logistic Regression	0.7085	0.5797	0.6456	0.0264	0.7019	0.7164	0.7483	0.6667
Random Forest	0.7559	0.6610	0.7032	0.0224	0.7500	0.7642	0.8165	0.6861
Cascade Classifier	0.8387	0.6923	0.7773	0.0343	0.8182	0.8539	0.8060	0.8636

Std: Standard deviation
